# Association of adrenal insufficiency with patient-oriented health-care outcomes in adult medical inpatients

**DOI:** 10.1530/EJE-19-0469

**Published:** 2019-10-03

**Authors:** Fahim Ebrahimi, Andrea Widmer, Ulrich Wagner, Beat Mueller, Philipp Schuetz, Mirjam Christ-Crain, Alexander Kutz

**Affiliations:** 1 Division of Endocrinology, Diabetes, and Metabolism, University Hospital Basel, Basel, Switzerland; 2 Division of Health and Social Affairs, Section Health, Swiss Federal Office for Statistics, Neuchâtel, Switzerland; 3 Division of General Internal and Emergency Medicine, University Department of Medicine, Kantonsspital Aarau, Aarau, Switzerland; 4 Division of Endocrinology, Diabetes, and Metabolism, University Department of Medicine, Kantonsspital Aarau, Aarau, Switzerland; 5 Faculty of Medicine, University of Basel, Basel, Switzerland; 6 Foundation National Institute for Cancer Epidemiology and Registration (NICER), University of Zurich, Zurich, Switzerland

## Abstract

**Objective:**

Adrenal insufficiency in the outpatient setting is associated with excess morbidity, mortality, and impaired quality of life. Evidence on its health-care burden in medical inpatients is scarce. The aim of this study was to assess the health-care burden of primary adrenal insufficiency (PAI) and secondary adrenal insufficiency (SAI) among hospitalized inpatients.

**Design and methods:**

In this nationwide cohort study, adult medical patients with either PAI or SAI hospitalized between 2011 and 2015 were compared with propensity-matched (1:1) medical controls, respectively. The primary outcome was 30-day all-cause in-hospital mortality. Main secondary outcomes included ICU admission rate, length-of-hospital stay, 30-day and 1-year all-cause readmission rates.

**Results:**

In total, 594 hospitalized cases with PAI and 4880 cases with SAI were included. Compared with matched controls, in-hospital mortality was not increased among PAI or SAI patients, respectively. Patients with adrenal insufficiency were more likely to be admitted to ICU (PAI: OR 1.9 (95% CI, 1.27 to 2.72) and SAI: OR 1.5 (95% CI, 1.35 to 1.75)). Length of hospital stay was prolonged by 1.0 days in PAI patients (8.9 vs 7.9 days (95% CI, 0.06 to 1.93)), and by 3.3 days in SAI patients (12.1 vs 8.8 days (95% CI, 2.82 to 3.71)), when compared with matched controls. Patients with SAI were found to have higher 30-day and 1-year readmission rates (14.1 vs 12.1% and 50.0 vs 40.7%; *P* < 0.001) than matched controls.

**Conclusions:**

While no difference in in-hospital mortality was found, adrenal insufficiency was associated with prolonged length of hospital stay, and substantially higher rates of ICU admission and hospital readmission.

## Introduction

Adrenal insufficiency is a potentially life-threatening disease and classified into primary adrenal insufficiency (PAI) and secondary adrenal insufficiency (SAI), respectively. In PAI steroid hormone production in the adrenal cortex is reduced (1, 2), whereas in patients with SAI, the disorder is at the level of the hypothalamic–pituitary–adrenal (HPA) axis (1, 3, 4, 5). If adrenal insufficiency is not recognized and substitution of in particular glucocorticoid hormones is not being initiated, the majority of patients will die within 2 years after diagnosis (6). While glucocorticoids help achieve sufficient quality of life (7), acute management of adrenal insufficiency in emergency situations especially among hospitalized patients remains a challenge in acute care institutions (8, 9).

Both, patients with PAI and patients with SAI, are known to have higher risks for metabolic and psychiatric comorbidities and their risk for hospital admission is elevated as compared to the general population (10).

Patients with SAI have an excess mortality, especially from vascular and respiratory disease (11). Similarly, the risk for death is more than two-fold increased among patients with PAI when compared with the general population (12). Excess mortality was highest when the patients were diagnosed at young age (13). Underlying reasons for the mortality gap were identified to being cardiovascular, malignant or infectious diseases (12, 14). Whether this association between PAI and infections is related to PAI itself or due to unphysiological adrenal replacement therapy is debated (14).

As most of the studies were done in the outpatient setting, evidence on health-care burden of either PAI or SAI among medical inpatients remains scarce. Herein, we investigated in a propensity-matched in-hospital population-based cohort the impact of adrenal insufficiency on patient-centered health care outcomes (i.e. 30-day all-cause in-hospital mortality, intensive care unit (ICU) admission, length of ICU stay, intubation rate, length of intubation, length of hospital stay (LOS), and all-cause readmission rates).

## Subjects and methods

### Participants, data sources, and study variables

In this nationwide cohort study, data were provided by the Swiss Federal Office for Statistics (Bundesamt für Statistik, Neuchâtel, Switzerland). In this database, the patient information is fully anonymized. No written informed consent was given to the patients who were unidentifiable due to pseudo-anonymization. The database includes all Swiss inpatient discharge records from acute care, general, and specialty hospitals, excluding hospital units of post-acute care institutions, regardless of payer, and thus, creates a near 100% sample of inpatient discharges in Switzerland between 2011 and 2015. Each patient in this database was identified uniquely so that re-hospitalizations could be tracked. A single patient may have more than one index admission in the study period. According to the SwissDRG definition, all admissions after 18 days from discharge or admissions into another hospital were defined as new case in the nationwide hospital claims data (15). For the endpoint of all-cause readmission rate, every re-hospitalization – either within 30 days or within 1 year, respectively – after discharge were counted as hospital readmission. Solely emergency admissions to hospital were included, whereas planned hospitalizations were excluded from the analysis. In patients who were transferred between acute care hospitals, the hospital stays were combined into a single episode of care and the patient outcome was attributed to the first hospitalization. The database included information such as patient’s residency, hospital teaching level, year and month of hospitalization, LOS as well as age at admission and admission diagnosis. Medical diagnoses were coded using the International Classification of Disease, version 10, German Modification (ICD-10 GM) codes (http://www.who.int/classifications/icd/en/). AIl hospitalizations were identified by applying (ICD-10-GM) code of E27.1 for PAI and E27.3 and E89.6 for SAI to primary or secondary discharge diagnoses, respectively. Patients without clear diagnosis of either PAI or SAI were not included in the analysis. Patients with tertiary adrenal insufficiency due to long-term administration of high doses of glucocorticoids were pooled with SAI patients. Nonmedical, psychiatric, and nonadult (<18 years of age) patients were excluded from the analysis.

The Institutional Review Board of Northwestern Switzerland approved this study and waived informed patient consent owing to the use of deidentified data. This study followed the Strengthening The Reporting of OBservational studies in Epidemiology (STROBE) reporting guideline (16).

### Outcomes

The primary outcome was 30-day all-cause in-hospital mortality. Secondary outcomes comprised ICU admission rate, length of ICU stay, intubation rate, length of intubation time, LOS – defined as days spent in the hospital during the hospitalization, as well as 30-day and 1-year all-cause readmission rates.

### Statistical analysis

Hospitalized cases meeting inclusion and exclusion criteria within each study cohort (PAI and SAI) were 1:1 propensity score matched to a general medical inpatient control group (matched control) using a nearest-neighbor matching algorithm. For optimal matching Hosmer-Lemeshow goodness of fit and concordance c-statistics were utilized to assess the fitness of the models. Furthermore, we used a threshold of 10% in the standardized difference as a meaningful imbalance between the two groups (17). Cases were matched based on age, sex, race/ethnicity, year of hospitalization, hospital teaching level, Charlson Comorbidity Index (CCI), admission diagnosis, and important comorbidities. Summary statistics were calculated for patient demographics and major comorbid conditions for each study cohort. Estimates of the effect size and corresponding 95% confidence intervals (CI) were determined using linear, logistica or Cox proportional hazards regression as appropriate. All *P* values are two-sided and have not been adjusted for multiple testing. Kaplan–Meier curves were used to illustrate differences in time to in-hospital death, time to discharge and time to readmission. All CIs are at the 95% level. Statistical analyses were performed using STATA, version 15.1 (StataCorp LLC).

## Results

### Patient characteristics

**Figure 1:**
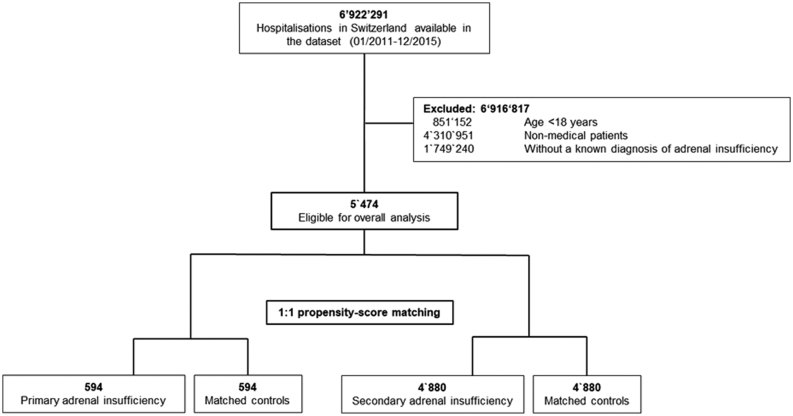
Flow chart of included hospitalized cases.

Between January 2011 through December 2015, we identified 6 922 291 hospitalizations of whom 5474 fulfilled eligibility criteria for matching. Of those, 594 hospitalized cases with PAI and 4880 cases with SAI were matched 1:1 with medical controls (Fig. 1). As designed, baseline characteristics were similar between both matched adrenal insufficiency cohorts (Table 1) without any significant differences. Among all study cohorts, median age was around 65 years and slightly more patients were female. Most key comorbid conditions were higher across patients with SAI when compared with PAI patients with higher rates of hypertension (47 vs 31%), renal insufficiency (31 vs 25%), chronic obstructive pulmonary disease (27 vs 12%), as well as cancer (22 vs 12%). Accordingly, patients with SAI had higher comorbidity indices shown by the CCI (2.4 vs 1.6 points). Causes of initial hospital admission were well matched among cases with PAI as well as SAI and their respective controls (Supplementary Fig. 1, see section on supplementary data given at the end of this article). Among cases with PAI, endocrine conditions were the predominant cause (25.9%) and infectious diseases the second prevalent cause (14.5%) of hospital admission. In contrast, among patients with SAI, respiratory conditions were the leading cause of hospital admission (21.4%), followed by oncologic/hematologic diseases (14.5%) (Supplementary Fig. 1).

**Table 1:** Baseline characteristics.

	Primary adrenal insufficiency vs matched controls			Secondary adrenal insufficiency vs matched controls		
	Primary adrenal insufficiency (*n* = 594)	Control (*n* = 594)	Standardized difference, %	Secondary adrenal insufficiency (*n* = 4880)	Control (*n* = 4880)	Standardized difference, %
Sociodemographics						
Age, mean (s.d.)	65.1 (19.0)	64.2 (20.2)	4.4	68.1 (14.9)	68.9 (16.2)	5.0
Female gender, *n* (%)	317 (53.4)	333 (56.1)	5.4	2447 (50.1%)	2519 (51.6%)	3.0
Swiss residents, *n* (%)	489 (82.3)	486 (81.8)	1.5	4133 (84.7%)	4164 (85.3%)	2.1
Hospital teaching level, *n* (%)						
Tertiary care hospital	407 (68.5)	424 (71.4)	6.2	3644 (74.7%)	3634 (74.5%)	0.5
Main reasons for hospital admission, *n* (%)						
Endocrine	154 (25.9)	149 (25.1)	1.9	550 (11.3)	505 (10.4)	3.0
Infections	86 (14.5)	88 (14.8)	0.9	559 (11.5)	537 (11.0)	1.4
Cardiovascular	79 (13.3)	70 (11.8)	4.6	548 (11.2)	502 (10.3)	3.0
Cancer	49 (8.2)	50 (8.4)	0.6	709 (14.5)	759 (15.6)	2.9
Pulmonary	76 (12.8)	96 (16.2)	9.6	1044 (21.4)	1113 (22.8)	3.4
Comorbidities, *n* (%)						
Diabetes mellitus	121 (20.4)	121 (20.4)	0.0	1147 (23.5)	1149 (23.5)	0.1
Hypertension	182 (30.6)	178 (30.0)	1.5	2290 (46.9)	2246 (46.0)	1.8
CAD	74 (12.5)	61 (10.3)	6.9	820 (16.8)	721 (14.8)	5.6
Cerebrovascular disease	22 (3.7)	19 (3.2)	2.8	180 (3.7)	164 (3.4)	1.8
Cancer	71 (12.0)	73 (12.3)	1.0	1076 (22.0)	1091 (22.4)	0.7
Renal insufficiency	150 (25.3)	150 (25.3)	0.0	1511 (31.0)	1518 (31.1)	0.3
COPD	72 (12.1)	76 (12.8)	2.0	1312 (26.9)	1291 (26.5)	1.0
Charlson comorbidity index, mean (s.d.)	1.6 (2.3)	1.6 (2.3)	1.1	2.4 (2.7)	2.5 (2.7)	0.9

CAD, Ccoronary artery disease; COPD, Cchronic obstructive pulmonary disease.

### Thirty-day all-cause in-hospital mortality

**Figure 2:**
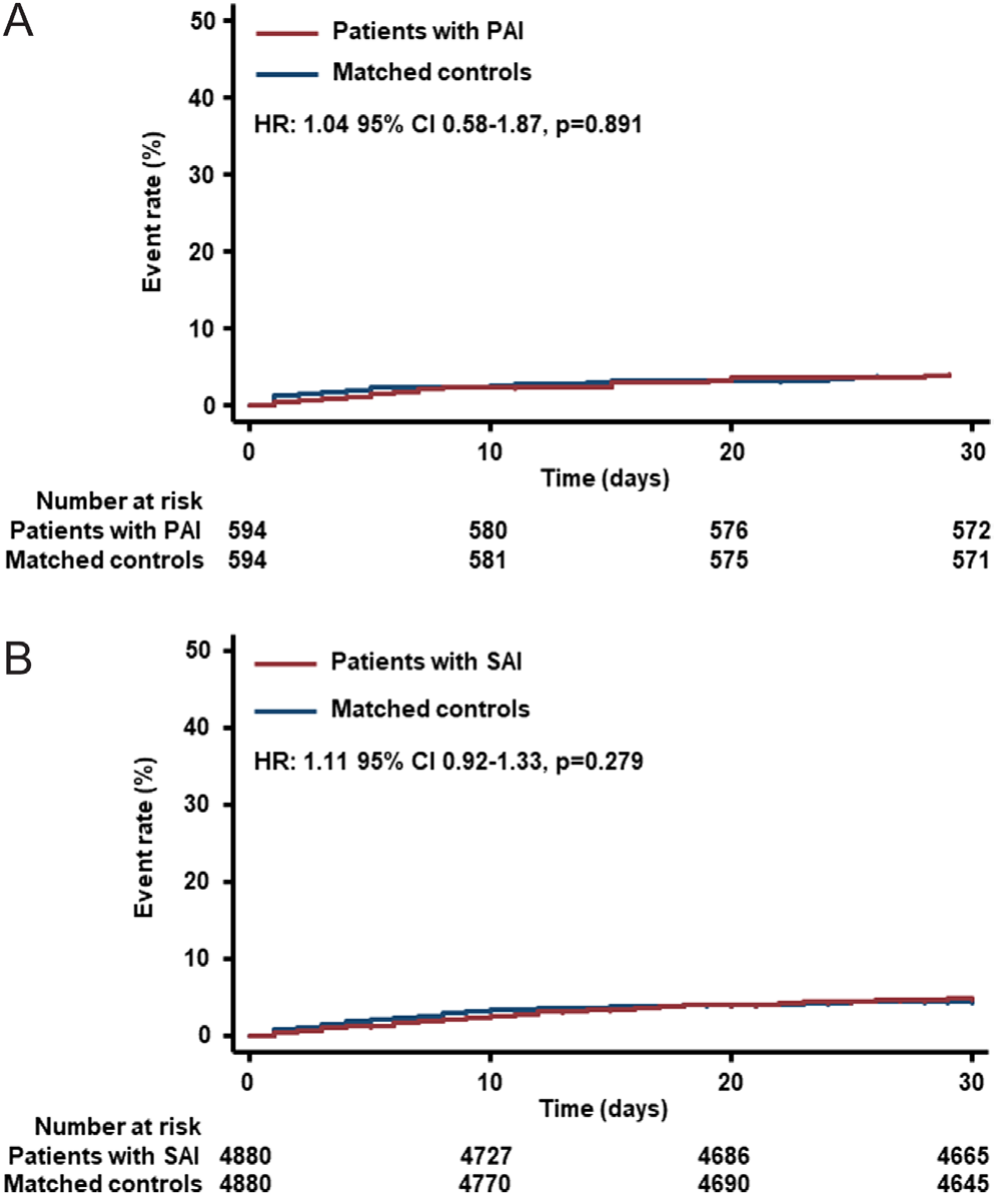
Thirty-day in-hospital mortality: (A) PAI cohort vs matched controls and (B) SAI cohort vs matched controls.

In-hospital mortality rates within 30 days were overall comparably low ranging from 3.9% (odds ratio (OR) 1.05, 95% CI 0.58 to 1.90) in the matched PAI cohorts to 4.9% (OR 1.12, 95% CI, 0.93 to 1.35) in the matched SAI cohorts (Table 2). Kaplan–Meier estimates revealed no differences in 30-day all-cause in-hospital mortality neither among patients with PAI (Fig. 2A), nor among patients with SAI (Fig. 2B) when compared with matched controls, respectively.

**Table 2:** Primary and secondary outcomes.

Patient outcomes	Comparison	*n* (%) *Duration (s.d.)	OR (95% CI) *Regression coefficient (95% CI)	*P* value
Primary outcome				
Thirty-day in-hospital mortality	Primary AI vs controls	23 vs 22 (3.9 vs 3.7)	1.05 (0.58 to 1.90)	0.879
	Secondary AI vs controls	240 vs 216 (4.9 vs 4.4)	1.12 (0.93 to 1.35)	0.250
Secondary outcomes				
ICU admission	Primary AI vs controls	80 vs 46 (13.5 vs 7.7)	1.85 (1.27 to 2.72)	0.002
	Secondary AI vs controls	647 vs 441 (13.3 vs 9.0)	1.54 (1.35 to 1.75)	<0.001
Length of ICU stay (hours)*	Primary AI vs controls	94.1 (166.6) vs 65.2 (86.6)	28.9 (−23.4 to 81.2)	0.277
	Secondary AI vs controls	129.6 (238.4) vs 88.9 (137.9)	40.7 (16.0 to 65.4)	0.001
Intubation	Primary AI vs controls	26 vs 17 (4.4 vs 2.9)	1.55 (0.83 to 2.89)	0.165
	Secondary AI vs controls	283 vs 187 (5.8 vs 3.8)	1.54 (1.28 to 1.87)	<0.001
Length of intubation (hours)*	Primary AI vs controls	137.9 (206.0) vs 44.6 (78.4)	93.3 (−12.6 to 199.3)	0.083
	Secondary AI vs controls	140.0 (262.9) vs 94.9 (142.1)	45.1 (3.82 to 86.39)	0.032
Length of hospital stay (days)*	Primary AI vs controls	8.9 (9.1) vs 7.9 (7.1)	0.99 (0.06 to 1.93)	0.037
	Secondary AI vs controls	12.1 (12.6) vs 8.8 (9.4)	3.27 (2.82 to 3.71)	<0.001
30-day readmission	Primary AI vs controls	52 vs 48 (8.8 vs 8.1)	1.09 (0.72 to 1.64)	0.676
	Secondary AI vs controls	690 vs 588 (14.1 vs 12.1)	1.20 (1.07 to 1.35)	0.002
1-year readmission	Primary AI vs controls	228 vs 209 (38.4 vs 35.2)	1.15 (0.91 to 1.45)	0.253
	Secondary AI vs controls	2439 vs 1987 (50.0 vs 40.7)	1.45 (1.34 to 1.58)	<0.001

*Data on length of ICU stay, length of intubation and length of hospital stay are presented as regression coefficients. All other outcomes are presented as odds ratios (OR).

### ICU admission and intubation

Patients with PAI had 1.9-fold increased odds of being admitted to ICU when compared with matched controls (OR 1.85, 95% CI, 1.27 to 2.72; *P* = 0.002). This observation was analog among patients with SAI (OR 1.54; 95% CI 1.35 to 1.75; *P* < 0.001). Length of ICU stay was not different between patients with PAI and matched controls, however, slightly increased by 1.7 days in patients with SAI compared to matched controls (Table 2). Among patients admitted to ICU, there was no significant difference in intubation rates in the PAI cohort compared to controls. However, for patients with SAI odds for intubation were 1.5-fold increased when compared to propensity score-matched controls (OR 1.54, 95% CI, 1.28 to 1.87; *P* < 0.001), with as well prolonged length of intubation (Table 2).

### Length of hospital stay and hospital readmission

**Figure 3:**
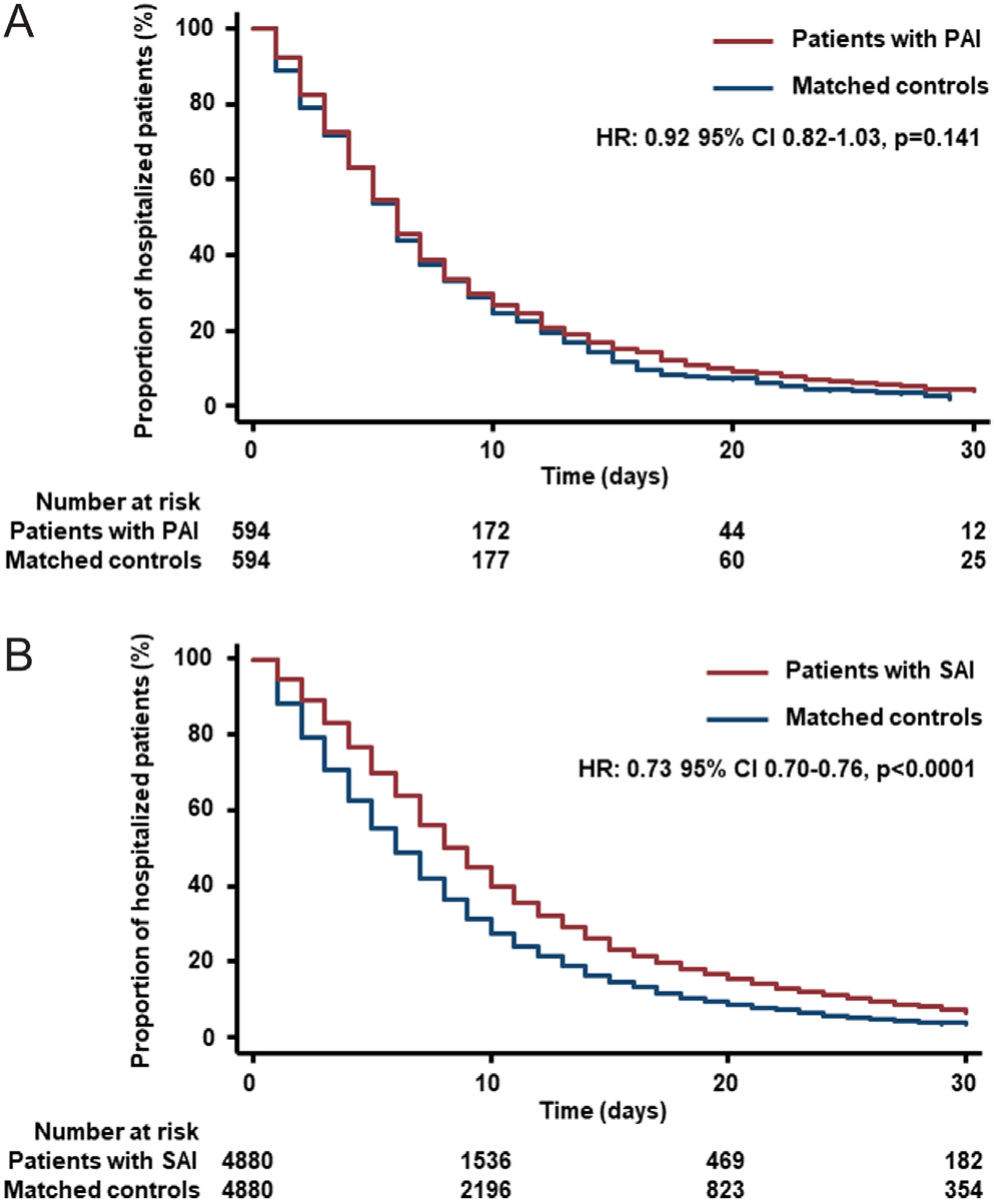
Time to discharge: (A) PAI cohort vs matched controls and (B) SAI cohort vs matched controls.

Compared with matched controls, mean LOS was prolonged by 1 day in PAI patients (95% CI, 0.06 to 1.93; *P* = 0.037) and by 3.3 days in SAI patients (95% CI, 2.82 to 3.71; *P* < 0.001) (Table 2). Accordingly, Kaplan–Meier estimates showed a longer time to discharge in both adrenal insufficiency groups compared to their corresponding matched cohorts (Fig. 3A and B).

**Figure 4:**
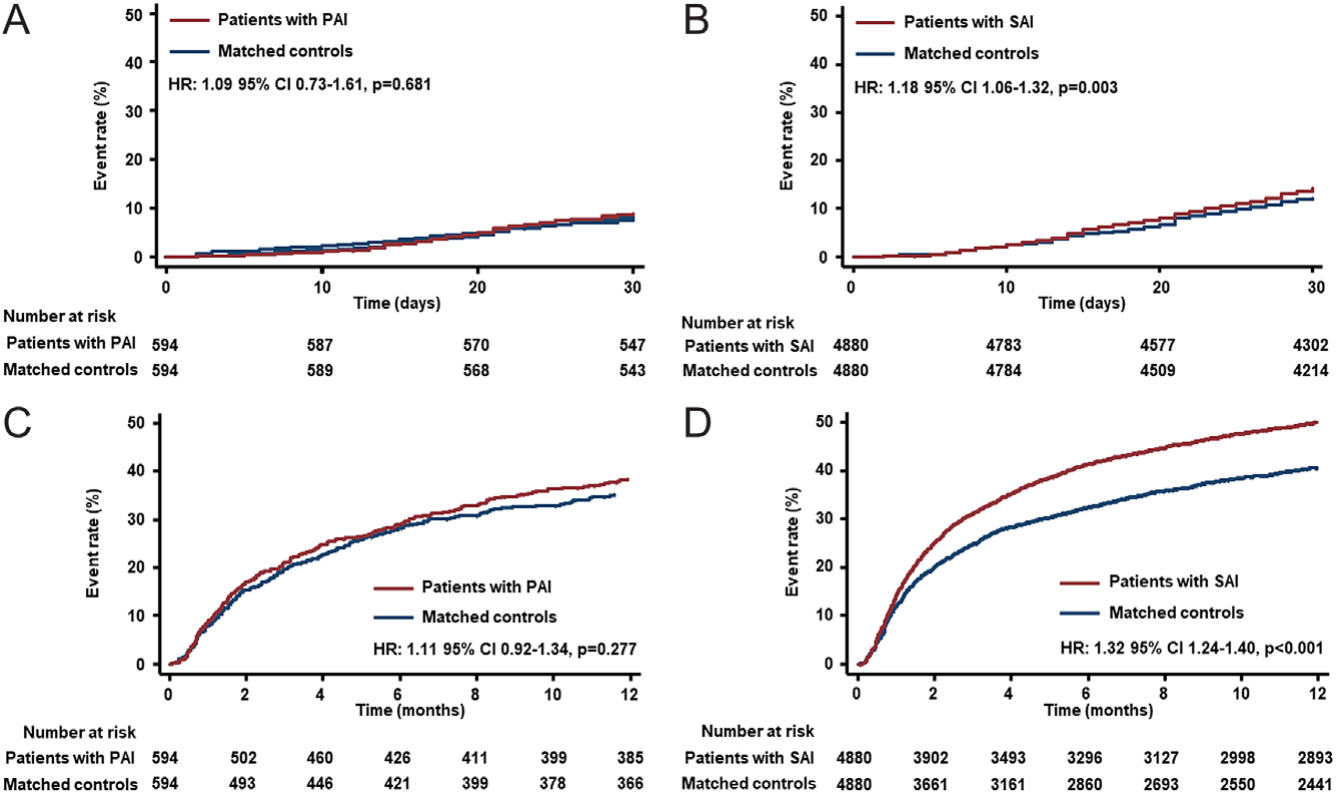
All-cause readmission rates: (A) 30-day- and (C) 1-year readmission rates in PAI cohort vs matched controls, and (B) 30-day and (D) 1-year readmission rates in SAI cohort vs matched controls.

In PAI patients there was no significant difference in 30-day all-cause hospital readmission in comparison with matched controls. However, 14.1% of SAI cases versus 12.1% of matched controls were readmitted to hospital within 30 days resulting in an odds ratio of 1.20 (95% CI, 1.07 to 1.35; *P* = 0.002) (Table 2). Corresponding Kaplan–Meier estimates are shown in Fig. 4A and B. At 1 year, there was no difference in hospital readmission rates among PAI patients when compared to matched controls, while half of the cases with SAI were re-admitted to hospital, compared with 40.7% among matched controls (OR 1.45, 95% CI 1.34 to 1.58; *P* < 0.001) (Fig. 4C, D and Table 2).

**Figure 5:**
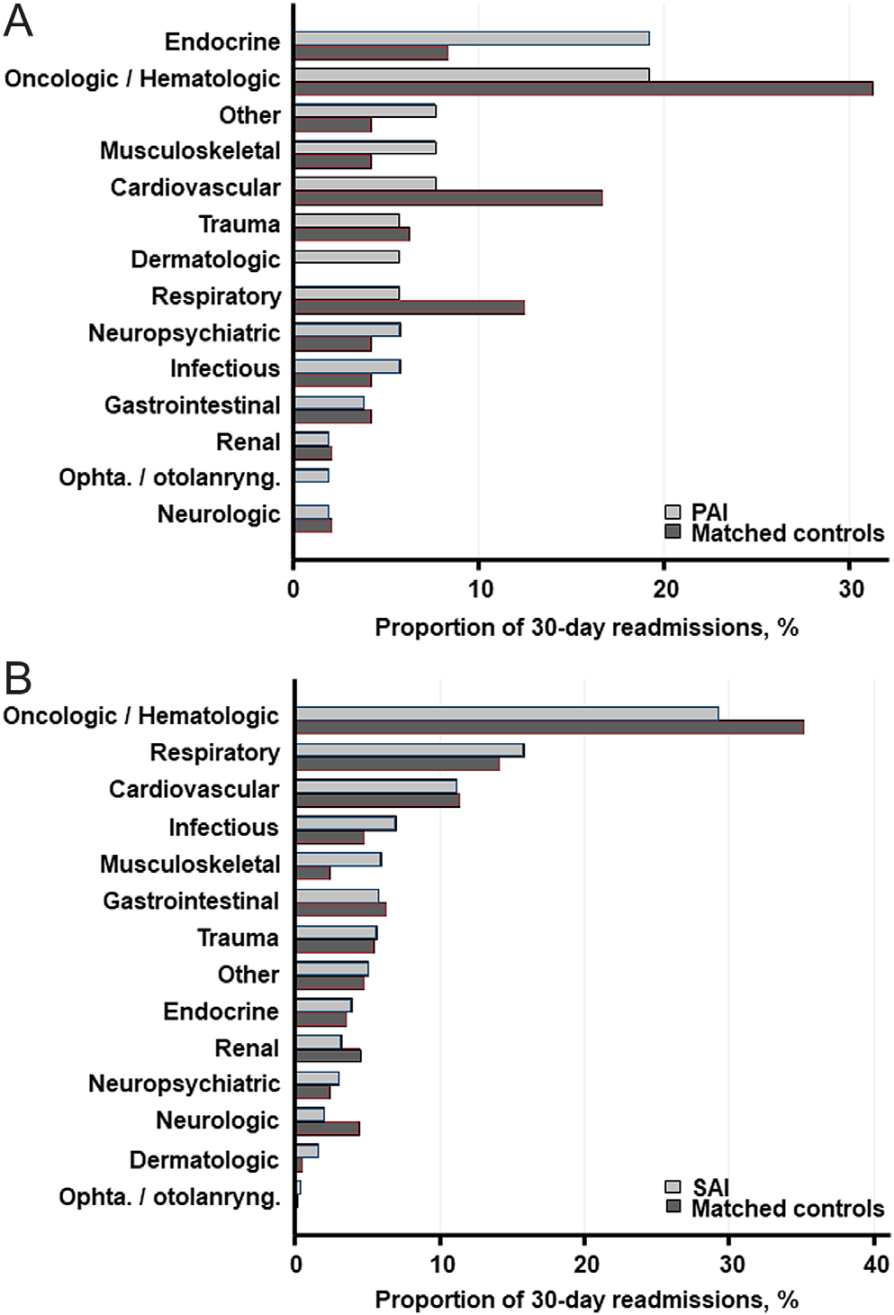
Causes of 30-day readmissions in patients with PAI (A) and SAI (B) versus their matched controls.

In hospitalized cases with PAI, 30-day readmissions were predominantly due to nonendocrine causes (80.8%) and in 19.2% due to endocrine causes. Compared with matched controls, patients with PAI had a numerically higher proportion of readmissions for endocrine reasons (10 (19.2%) vs 4 (8.3%)) and higher proportion of musculoskeletal (4 (7.7%) vs 2 (4.2%)), and dermatologic (3 (5.8%) vs 0) causes of readmissions; however, none of these differences met statistical significance (Fig. 5A and Supplementary Table 1).

Of the 690 30-day readmissions in cases with SAI, 663 (96.1%) were due to nonendocrine causes and 27 (3.9%) were due to endocrine causes. Among nonendocrine causes of readmission, respiratory (109 (15.8%) vs 83 (14.1%)), infectious (48 (7.0%) vs 28 (4.8%)), and musculoskeletal (41 (5.0%) vs 14 (2.4%)) readmissions were numerically more prevalent in patients with SAI compared to matched controls, however, merely the latter meeting statistical significance (Fig. 5B and Supplementary Table 1). The most common cause, in general, was oncologic and hematologic, but significantly more prevalent among matched controls (202 (29.3%) vs 207 (35.2%), *P* = 0.02) (Fig. 5B).

## Discussion

This population-based cohort study assessing the health-care burden of more than 5000 propensity score-matched hospitalized cases with adrenal insufficiency has two key findings: First, adrenal insufficiency carries a clinically relevant health-care burden, as mirrored by increased ICU admission and intubation rates, prolonged LOS, and higher hospital readmission rates, in particular among cases with SAI. Second, it is nonetheless reassuring that unlike data on long-term survival in the outpatient setting (12), there was no increased in-hospital mortality in our study. Whether our findings are due to higher demand of health-care resources as mirrored by prolonged hospital stay and increased readmission rates or due to higher vulnerability of patients with adrenal insufficiency remains debatable.

In line with our findings, outpatients with adrenal insufficiency are at increased risk of hospital admission and reduced quality-of-life when compared with the general population (10, 18, 19). Due to long-term excess of glucocorticoid exposure, patients with adrenal insufficiency are at increased risk of developing comorbid conditions such as obesity, diabetes mellitus, hypertension, and depression (10). These comorbidities strongly contribute to an increased cardiovascular risk profile (20, 21). Against this background we used a propensity score matching taking all relevant and documented comorbidities into account achieving well-balanced patient characteristics when compared to matched controls, in order to minimize possible confounding on the investigated outcomes.

Worse outcomes in patients with adrenal insufficiency may at least in part be explained by an inappropriate glucocorticoid replacement therapy in the context of stress and acute illnesses. In fact, previous studies have revealed that doses of glucocorticoid replacement were often too high and should be reduced in around half of the patients (22). Besides, glucocorticoid hormone supplementation in the acute care setting is generally complicated by the need to adjust glucocorticoid dosage during hospitalization which may as well explain a prolonged hospital stay and higher demand of health-care resources (23).

In addition, there is increasing evidence that patients with adrenal insufficiency on glucocorticoid replacement therapy often exhibit a pro-inflammatory state and weakened immune defense due to over-supply, which has recently been shown to be less pronounced with modified-release preparations when compared with multiple daily dosing of short-acting formulations (24). Consequently, patients with adrenal insufficiency are known to suffer from more frequent and severe infections (14), mainly due to an impairment of innate and adaptive immunity (25). On the other hand, under-supply can lead to adrenal crisis and reduced quality of life (9, 26). Hence, observed differences in ICU admission rates and LOS may therefore be due to an augmented susceptibility to hemodynamic instability in the context of acute medical conditions, such as infections, trauma, and major psychological distress which are known predisposing factors for adrenal crisis (9, 27, 28). In agreement with the literature, we found a numerically higher amount of infections and cardiovascular reasons as main cause for hospital readmissions in SAI patients compared to matched controls, however, not meeting statistical significance, respectively. Interestingly, the main causes for 30-day hospital readmission did not relevantly differ from matched controls, neither in PAI nor in SAI patients. This further supports the robustness of the matched cohorts and the validity of the model considering index admission causes which have been described among the main determinants of readmission causes (29).

It is worth mentioning that patients with tertiary adrenal insufficiency were pooled into the SAI cohort which may have pertinently influenced the results since these patients have high rates of oncologic, respiratory, and cardiovascular diagnoses. Since many patients in the SAI cohort had iatrogenic adrenal insufficiency from high-dose glucocorticoid treatment, there was in fact a higher rate of COPD and cancer among SAI patients. However, due to common ICD coding of secondary and tertiary adrenal insufficiency in the SwissDRG dataset, subgroup analyses investigating differences in outcomes between these patient cohorts were not feasible.

Nevertheless, challenging our findings one could hypothesize that the higher ICU admission rates and prolonged LOS do not reflect the burden of disease *per se* but rather a higher awareness of physicians toward the vulnerability of patients with adrenal insufficiency. However, current literature does not provide sufficient evidence for this hypothesis (23).

Our study has some limitations. First, the administrative nature of the database and the cross-sectional design carry the risk for unmeasured confounding, under-reporting of secondary diagnoses, and varying accuracy of coding between hospitals. Furthermore, information bias in coding of patients with underlying chronic conditions cannot be excluded in discharge claims data. Second, lack of data on etiology and duration of SAI as well as on glucocorticoid supplementation did not allow for cluster analyses, respectively. Importantly, there may have been patients with pituitary disorders and dysfunction of other pituitary axes which could have had an effect modification on the assessed outcomes. Third, propensity score matching could not account for the severity of the acute medical condition leading to hospitalization. Finally, our analysis was limited to hospitalized patients with acute medical conditions. Episodes of adrenal insufficiency diagnosed and treated in emergency departments but not hospitalized were not available and therefore missing in our study. Thus, the findings may not be generalizable to patients admitted to surgical departments, or other specialties, and as well not to outpatient care of patients with adrenal insufficiency.

However, the following strengths are noteworthy: we used registry data spanning nationwide hospital care with a large sample size, high representativity, and a long study period. Thus, with the application of a weighted analysis, our study reflects highly accurate estimates at the national level. As these national estimates have not been presented before, it would be useful to replicate this study using different and more sophisticated resources.

In conclusion, there is a relevant health-care burden among adult patients with adrenal insufficiency hospitalized for acute medical conditions. Based on these findings increased efforts are warranted to tackle these short-comings of patient-oriented outcomes in this vulnerable patient population.

## Supplementary Material

Supplemental Figure S1 Causes of index hospital admission in patients with PAI (A) and SAI (B) versus their matched controls.

Supplementary Table S1 – Causes of 30-day readmissions

## Declaration of interest

The authors declare that there is no conflict of interest that could be perceived as prejudicing the impartiality of this study.

## Funding

This study was supported in part by the Swiss National Science Foundation (SNSF, National Research Program (NRP 74), 407440_167376).

## Author contribution statement

F E, A W, M C C, and A K designed the study and wrote the manuscript. A K had access to all the data. F E and A K analyzed the data and were responsible for the decision to submit the manuscript. All authors provided comments on drafts and approved the final report.

## Role of the funding source

The funding sources had no role in study design, data collection, data analysis, data interpretation, or writing of the report.
