# Supplementary figures and images for: Association of adrenal insufficiency with patient-oriented health-care outcomes in adult medical inpatients
Source: Eur J Endocrinol. 2019 Oct 3;181(6):701–9. doi: 10.1530/EJE-19-0469 (PMC6977938; doi:10.1530/EJE-19-0469)

Supplemental Figure 1

A

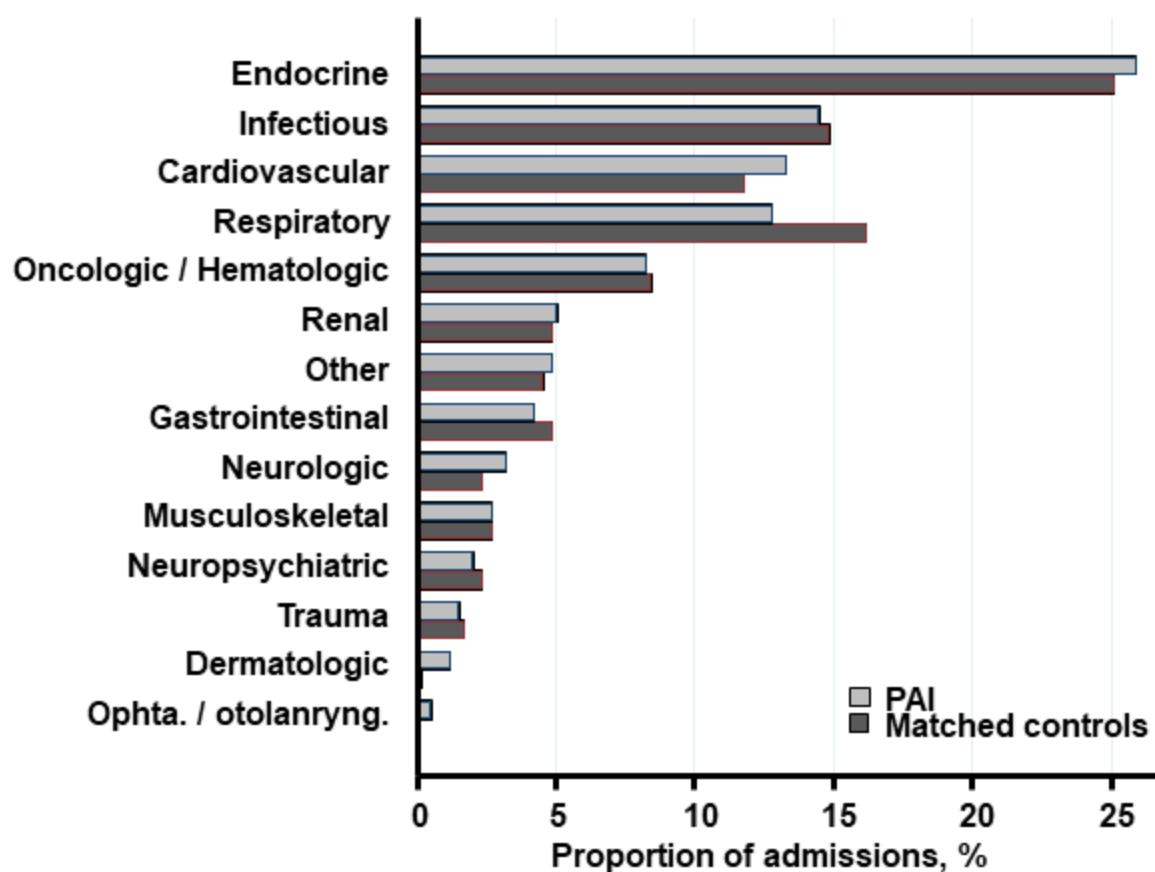

B

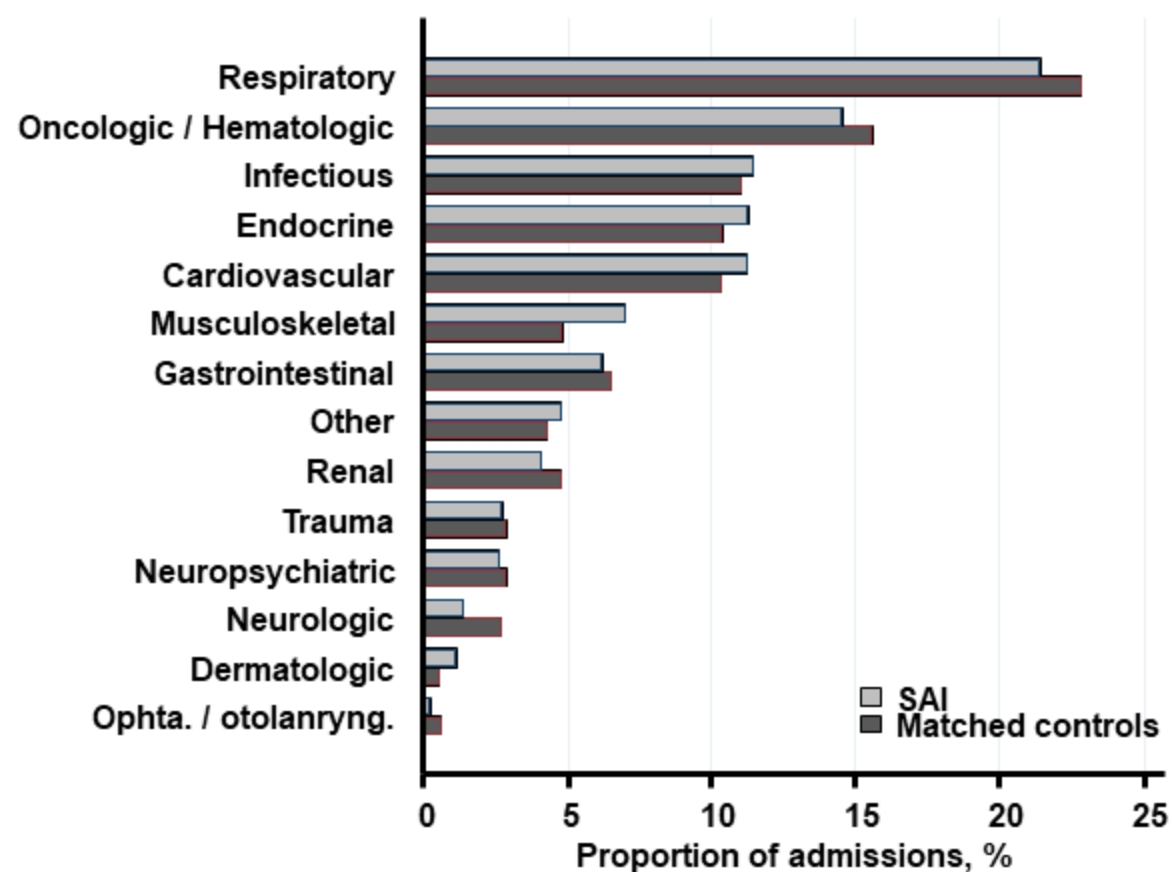

Supplement: Supplemental Figure S1 Causes of index hospital admission in patients with PAI (A) and SAI (B) versus their matched controls. [file supplementary_figure_1.pdf]
