# Supplementary material for: Association of adrenal insufficiency with patient-oriented health-care outcomes in adult medical inpatients
Source: Eur J Endocrinol. 2019 Oct 3;181(6):701–9. doi: 10.1530/EJE-19-0469 (PMC6977938; doi:10.1530/EJE-19-0469)
Supplement: Supplementary Table S1 – Causes of 30-day readmissions [file supplementary_table_1.pdf]

Supplementary Table S1 – Causes of 30-day readmissions

Causes for 30-day readmissions	Comparison	n (%)	p-value
Infections	PAI vs controls	3 vs 2 (5.8 vs 4.2)	0.72
	SAI vs controls	48 vs 28 (7.0 vs 4.8)	0.10
Cancer / hematologic	PAI vs controls	10 vs 15 (19.2 vs 31.3)	0.17
	SAI vs controls	202 vs 207 (29.3 vs 35.2)	0.02
Endocrine	PAI vs controls	10 vs 4 (19.2 vs 8.3)	0.12
	SAI vs controls	27 vs 21 (3.9 vs 3.6)	0.75
Neuropsychiatric	PAI vs controls	3 vs 2 (5.8 vs 4.2)	0.72
	SAI vs controls	14 vs 26 (2.0 vs 4.4)	0.01
Neurologic	PAI vs controls	1 vs 1 (19.2 vs 20.8)	0.96
	SAI vs controls	21 vs 14 (3.1 vs 2.4)	0.47
Ophtalmologic / otolanryngologic	PAI vs controls	1 vs 0 (1.9 vs 0.0)	0.34
	SAI vs controls	3 vs 1 (0.4 vs 0.2)	0.40
Cardiovascular	PAI vs controls	4 vs 8 (7.7 vs 16.7)	0.17
	SAI vs controls	77 vs 67 (11.2 vs 11.4)	0.90
Respiratory	PAI vs controls	3 vs 6 (5.8 vs 12.5)	0.24
	SAI vs controls	109 vs 83 (15.8 vs 14.1)	0.40
Gastrointestinal	PAI vs controls	2 vs 2 (3.8 vs 4.2)	0.94
	SAI vs controls	40 vs 37 (5.8 vs 6.3)	0.72
Dermatological	PAI vs controls	3 vs 0 (5.8 vs 0.0)	0.09
	SAI vs controls	11 vs 3 (1.6 vs 0.5)	0.06
Musculoskeletal	PAI vs controls	4 vs 2 (7.7 vs 4.2)	0.46
	SAI vs controls	41 vs 14 (6.0 vs 2.4)	<0.01
Renal	PAI vs controls	1 vs 1 (1.9 vs 2.1)	0.96
	SAI vs controls	22 vs 27 (3.2 vs 4.6)	0.19
Others	PAI vs controls	4 vs 2 (7.7 vs 4.2)	0.46
	SAI vs controls	35 vs 28 (5.1 vs 4.8)	0.79
Trauma	PAI vs controls	3 vs 3 (5.8 vs 6.3)	0.92
	SAI vs controls	39 vs 32 (5.7 vs 5.4)	0.87

PAI, primary adrenal insufficiency; SAI secondary adrenal insufficiency.
